# Identification and Profiling of Pituitary microRNAs of Sheep during Anestrus and Estrus Stages

**DOI:** 10.3390/ani10030402

**Published:** 2020-02-29

**Authors:** Yaseen Ullah, Cunyuan Li, Xiaoyue Li, Wei Ni, Rui Yao, Yueren Xu, Renzhe Quan, Huixiang Li, Mengdan Zhang, Li Liu, Ruirui Hu, Tao Guo, Yaxin Li, Xiaokui Wang, Shengwei Hu

**Affiliations:** 1 College of Life Sciences, Shihezi University, Shihezi 832003, China; hbksons06@yahoo.com (Y.U.); 18738595903@163.com (C.L.); 18799297836@163.com (X.L.); 15739330255@163.com (R.Y.); 15136705752@163.com (Y.X.); 15509935295@163.com (R.Q.); 15739333350@163.com (H.L.); mdzzhang@163.com (M.Z.); 15609936261@163.com (L.L.); hrr1581268688@163.com (R.H.); guoxiaotao0212@163.com (T.G.); l787807273@163.com (Y.L.); wxk13179938152@163.com (X.W.); 2 College of Animal Science and Technology, Shihezi University, Shihezi 832003, China

**Keywords:** microRNAs, sheep, pituitary gland, expression profiles, estrus, anestrus

## Abstract

**Simple Summary:**

Sheep have an indispensable position in the world. Studies have shown that the reproduction of sheep is regulated by the photoperiod. However, sheep are short-lived animals that often rub in the fall. MicroRNAs are a class of non-coding RNAs of 21 to 25 nucleotides that play an important role in animal development. The expression and role of miRNAs in the estrus regulation of sheep are unclear. In this study, we present miRNA expression profiles in the sheep pituitary gland in the estrus and anestrus states. We detected a total of 199 miRNAs and 25 differentially expressed miRNAs in sheep pituitary gland during estrus and anestrus states. Six miRNAs were examined by reverse transcription quantitative-PCR and were significantly differentially (*p* < 0.05) expressed during the anestrus and estrus stages. The KEGG pathway and GO analysis have considerably augmented some of the miRNAs that play a key role in regulating basic biological processes. Our research offers valuable understanding into miRNA biology and aids to comprehend the purpose of miRNA in regulating major biological changes in animals.

**Abstract:**

MicroRNAs (miRNAs) are a class of small non-coding RNAs, molecules of 21 to 25 nucleotides in length, that regulate gene expression by binding to their target mRNA and play a significant role in animal development. The expression and role of miRNAs in regulating sheep estrus, however, remain elusive. Transcriptome analysis is helpful to understand the biological roles of miRNAs in the pituitary gland of sheep. A sheep’s pituitary gland has a significant difference between estrus and anestrus states. Here, we investigate the expression profiles of sheep anterior pituitary microRNAs (miRNAs) in two states, estrus and anestrus, using Illumina HiSeq-technology. This study identified a total of 199 miRNAs and 25 differentially expressed miRNAs in the estrus and anestrus pituitary gland in sheep. Reverse transcription quantitative-PCR (RT-qPCR) analysis shows six differentially (*p* < 0.05) expressed miRNAs, that are miR-143, miR-199a, miR-181a, miR-200a, miR-218, and miR-221 in both estrus and anestrus states. miRNAs containing estrus-related terms and pathways regulation are enriched using enrichment analysis from gene ontology (GO) and the Kyoto Encyclopedia of Genes and Genomes (KEGG). Moreover, we also envisioned a miRNA–mRNA interaction network to understand the function of miRNAs involved in the pituitary gland regulatory network. In conclusion, miRNA expression profiles in sheep pituitary gland in the anestrus and estrus deliver a theoretical basis for the study of pituitary gland biology in sheep.

## 1. Introduction

MicroRNAs are a class of small non-coding RNA molecules, of about 21–25 nucleotides in length that regulate gene expression by base pairing with their target mRNAs, primarily leading to protein inhibition. Initially, genes encoding miRNA in the nucleus are converted into primary transcripts by RNA polymerase II [1]. Next, primary transcripts are processed by RNase III Drosha enzyme, generating about 70 nucleotides long precursor molecules of miRNA. Finally, these precursor molecules of miRNA are conveyed from the nucleus to cytoplasm where they are further treated by RNase III protein Dicer into a 21–25 nucleotides miRNA duplex [2]. After the discovery of the first miRNA lin-4 in the pathogen of C. elegans (Caenorhabditis elegans) in 1993, miRNAs were recognized in plants, animals, and viruses [3]. Lin-4 was recognized to determine the development time of C. elegans larvae. Nevertheless, the miRNA was not documented as a conserved class of organic regulators until a second miRNA, let-7, was known. In recent decades, thousands of miRNAs have been identified in Caenorhabditis elegans, Homo sapiens, Drosophila melanogaster, Arabidopsis thaliana, Mus musculus, and Danio rerio. They have various biological functions that control cell proliferation, cell death, lipid metabolism, the fate of nerve cells, hormone secretion, and other stages of disease [4]. miRNAs are mostly present in tissue-specific patterns [5]. One miRNA can bind many mRNAs, while the target mRNA can be regulated by hundreds of miRNAs. These miRNAs can control a variety of biological functions, such as cell division, cell death, fat metabolism, hormone secretion, and tumorigenesis [6,7,8]. MiRNAs mainly target mRNA by binding to its 3′ untranslated region (3-UTR). However, miRNAs have also been shown to bind the coding sequence and to the 5′ untranslated region (5-UTR) [9,10].

The pituitary gland can be considered as the “master” endocrine organ of the body, acting as the central endocrine regulator of animal growth, bone metabolism, and the cell generation cycle [11]. It takes its signal from the brain and utilizes these messages to yield hormones. In particular, the anterior pituitary organ plays an important role in vertebrates’ homeostatic regulation. It executes its activities by releasing various hormones through five types of cells, including (a) corticotrophs secreting ACTH (adrenocorticotropic hormone), (b) thyrotrophs secreting TSH (thyroid-stimulating hormone), (c) gonadotrophs secreting LH (luteinizing hormone) and FSH (follicle-stimulating hormone), (d) somatotrophs secreting GH (growth hormone), and (e) lactotrophs secreting PRL (prolactin) [12]. Among them, LH (luteinizing hormone) and FSH (follicle-stimulating hormone) play an important role in the estrus state and anestrus state of animals [13] at this point.

In recent years, miRNAs, which are essential for the development of the anterior pituitary gland, have been studied step by step. For example, in mice miR-26b regulates the expression of Pit-1 (pituitary-specific positive transcription factor 1) by inhibiting Lef-1 (lymphoid enhancer factor 1) expression. Lef-1 is a key transcription factor that is involved in many growth processes. It is a sequence-specific DNA-binding protein that is expressed in pre-B and T lymphocytes of adult mice [14]. Lef-1 may promote differentiation of Pit-1 lineage during the development of the pituitary gland [12]. In the rat pituitary gland, miR-325-3p targets LHβ and inhibits LH synthesis and secretion [15]. Pit-1 (pituitary-specific positive transcription factor 1), also known as GHF-1 (growth hormone factor 1), is a specific pituitary transcription factor involved in mammalian pituitary development and hormone expression [16].

POMC (pro-opiomelanocortin) is a precursor of the pituitary melanocortin-related peptide and is regulated primarily by CRF (corticotropin-releasing factor). Previous studies revealed that miR-375 is greatly expressed in mice pituitary and that overexpression of miR-375 negatively regulates CRF-stimulated POMC expression and ACTH secretion [17]. All of these reports recommend that miRNAs could be an important regulator in sheep pituitary hormone secretion. miRNAs may play a substantial role in perpetuating the morphological structure of the intestine and in controlling its anatomical functions. Studies have shown that miRNAs are extensively expressed in the intestinal tissues of bovine, rodent, and swine. Previous studies on miRNAs have been performed in various tissues of sheep such as fat, heart, muscle, ovary, and hair follicles [18]. miRNAs may be involved in natural progression as well as in response to damage in cardiovascular conditions; most likely, they can be used as diagnostic biomarkers and therapeutic targets in hearth disease especially, myocardial infarction [19]. Several studies have linked 157 genes to major pituitary growth pathways and have identified 222 miRNAs in the pituitary glands that may influence growth after birth in pigs [20]. There are still clusters of different miRNA families found in humans and other related species not yet discovered in cattle. Currently, 2588 miRNAs of human and 1915 miRNAs from mouse genomes are deposited in miRBasev21 [21]. The involvement of miRNAs in sheep muscle development was recently reported [22].

To the best of our knowledge, the role of miRNAs in sheep seasonal estrus regulation and mode of expression in the pituitary is still unclear. Studies have shown that sheep reproduction is regulated by the photoperiod [23]. Estrus is a phase of increased female sexual receptivity, proceptivity, selectivity, and attractiveness [24]. On the other hand, anestrus is a period of complete reproductive incompetence, marked by reduction in hypothalamic GnRH (Gonadotropin-releasing hormone) content and secretion with a substantial reduction in the secretion of LH and FSH from the pituitary gland [25]. Sheep’s seasonal estrus usually occurs in the fall (September–February), followed by the anestrus. The average estrus cycle in sheep is about 16–17 days and the complete estrus cycle in sheep takes approximately 18 days [26].

Ovine estrus regulations play an important role in growing meat and wool production in sheep commerce. According to reports, regular feeding causes estrus and therefore anestrus in sheep with low feeding status [4].

Kazakh sheep in northern China are known to both breeders and consumers due to its quality meat and high adjustability. However, the anestrus period in these sheep is usually very long from April to July. Therefore, during anestrus season ewes are unable to mate and lambs are not available for quite a few months [27].

In the past few years, the usage of Illu-HiSeq sequencing of the transcriptome has increased with the prospect of classifying differential expression and discovering new miRNAs. In order to fully explore and understand the role of miRNAs in pituitary regulation, we determined miRNA expression profiles in sheep in estrus (E) and anestrus (A) phases by Illu-HiSeq sequencing, assuming that there are differentially expressed miRNAs in these two physiological states. These data will help us to understand the role of miRNAs in the regulation of seasonal fertility, pituitary growth, and development in animals.

## 2. Materials and Methods

### 2.1. Ethical Statement

All experiments involving animals were carried out under the regulations approved by the Animal Care and Use Committee of Shihezi University (SU-ACUC-08032).

### 2.2. Sample Collection and Preparation

Healthy Kazakh ewes used in this study lived in the natural pasture of Shihezi, Xinjiang, China. Six ewes, about two years old, weighing 45 kg were selected. Three ewes were selected during the estrus season (September; Xinjiang, China), and three ewes were selected during the anestrus season (April; Xinjiang, China).

One month before estrus, rams and ewes were kept together for no less than 10 min daily. Twice a day estrus was monitored according to the well-known estrus sign by experts.

Once the back pressure test was carried out the sheep showed a rigid stationary state, the vulva turned red and extruded, and clear yellow mucus released from the vulva, which is a sign of estrus period; these sheep were part of the estrus pituitary group (PGE). During our first estrus observation on the first day of the estrus cycle, three sheep were selected for slaughter in order to collect the anterior pituitary gland. On the other hand, if there is no evidence of estrus after 34–36 days of second estrus cycles, it is called anestrus period [26]. At this stage, three ewes were slaughtered to collect the anterior pituitary, and designated as the anestrus pituitary group (PGA). The pituitary samples were directly frozen in liquid nitrogen and preserved at −80 °C for later use followed by total RNA extraction. Independent t-test was performed to establish two groups composed of 3 sheep.

### 2.3. Total RNA Extraction and Small RNA Sequencing

According to the manufacturer’s instructions, the pituitary tissues of sheep estrus and anestrus were homogenized independently in liquid nitrogen, and total RNA was extracted using Trizol method (Cat. # 15596-026; Invitrogen, Carlsbad, CA, USA).

The quantity and quality of total RNAs were confirmed by the Bioanalyzer 2100 system and the RNA 6000 Nano kit (Cat. # DP315; Agilent Technologies, Santa Clara, CA, USA). After testing the sample, a small RNA library and high-throughput sequencing, three samples of both PAG and PGE were sequenced independently by Beijing Nuowheyuan Bioinformatics Co., Ltd. Subsequently, a sequencing library was made using the NEBNext Super Direction RNA Illumina (NEB, Ipswich, MA, USA), following the manufacturer’s recommendations. Using the specific structure of 3’and 5’ end of sRNA, total RNA was used as an initial sample to be ligated at both ends of the sRNA and reverse transcribed into cDNA. Then, the target DNA fragment was isolated by gel electrophoresis and the gel was recovered for the cDNA library. After pooling, RNA-Seq sequencing was performed according to the requirements for effective concentration and target data. 

### 2.4. Bioinformatics Analysis of Small RNA Sequences

**Figure 1:**
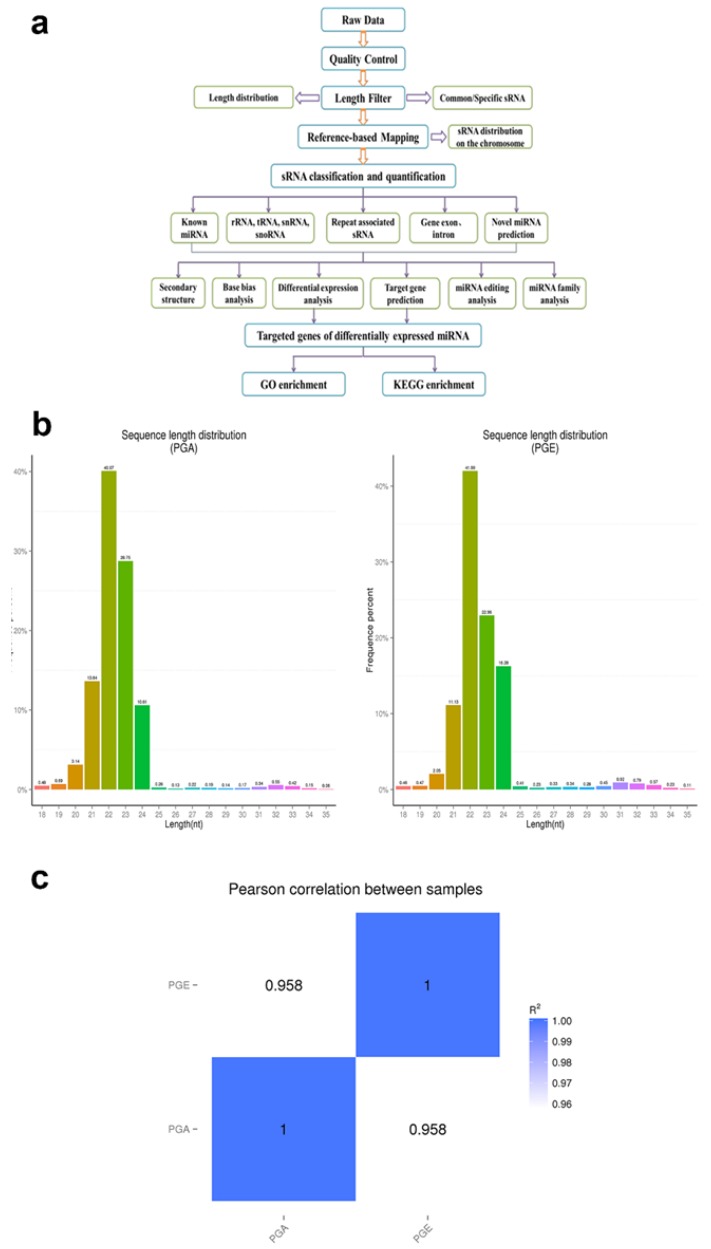
Identification and classification of miRNAs in sheep pituitary. (**a**) Workflow for identifying miRNAs. (**b**) The length distribution of sequence small RNAs in the clean read. (**c**) Schematic diagram of Pearson’s correlation of miRNA expression levels between samples; horizontal axis is the length of miRNA and the vertical axis indicates the abundance of the miRNA. PGA, anestrus pituitary group; PGE, estrus pituitary group.

Statistical analysis of the length of sRNA was performed and sRNA shorter than 18 nucleotides were filtered for successive analysis. Types of sRNA are determined by the length of the distribution peak. Gene annotation and reference genome were downloaded from sheep reference genome [26]. Using Bowtie software, the sRNA was mapped to a reference sequence. The sequence was then compared to the RepeatMasker database and non-coding sRNAs such as rRNA, snoRNA, snRNA, and tRNA were deleted. The remaining sRNAs were compared to miRBase 20.0 to discover known miRNAs and novel miRNAs were identified using miREvo [28] and mirdeep2 software [29]. The expression levels of identified miRNAs were analyzed using TPM (transcripts per million; miRNAs normalized by TPM) [30]. TPM density distribution analysis was achieved to carefully investigate the gene expression patterns of these miRNAs. For the purpose of screening differentially expressed miRNAs, the threshold was set as q-value < 0.01 and |log2 (foldchange)|> 1 by default. The miRNA analysis was performed according to the flow chart (Figure 1a).

### 2.5. Real-Time Quantitative Reverse Transcription PCR Analysis

Total RNA was extracted from sheep pituitary using RNA Kit (Cat. # 74106; Qiagen, Germany). cDNA was synthesized from the purified 500 ng total RNA using reverse transcription PCR (RT-PCR) Kit (Takara, Japan). RT-PCR was performed using specific primers for six miRNAs (miR-143, miR-199a, miR-181a, miR-200a, miR-218, and miR-221). RT-PCR amplification products were examined by electrophoresis and DNA sequencing to confirm the presence of miRNAs. Reverse transcription quantitative-PCR (RT-qPCR) was used to verify differential expression. All miRNAs identified according to the manufacturer’s protocol were subjected to RT-qPCR analysis using SYBR Green (Takara). RT-qPCR was performed using the following conditions: Initial denaturation at 94 °C for 10 min, subsequently 40 cycles at 55 °C for 30 s, 55 °C for 10 s, and 72 °C for 32 s, using a LightCycler 480 II system (Roche, Mannheim, Germany). 

U6 was used as a core reference to normalize miRNA expression. Three independent biological replicates were performed for all samples of qPCR. Expression levels of these genes were determined using SYBR Green Real-Time PCR. Next, the data were analyzed using the formula 2ΔΔCt. Primers are listed in Table S1.

### 2.6. Gene Prediction and Target Enrichment Analysis

MiRNAs bind to the 3-UTR (untranslated region) of the target mRNA, destabilize the mRNA, and perform translational silencing to suppress protein production [31]. To better understand the mechanism by which miRNAs bind to mRNA, we used miRanda software to predict miRNA–mRNA interaction networks.

The results were presented by cytoscape 3.7.2. Encyclopedia of Genes and Genomes (KEGG) enrichment analysis [32] and the International Standard Classification System for Gene Pathways in Gene Ontology (GO) Analysis [26,33] were used to perform enrichment analysis of differentially expressed miRNAs of two states. Host genes of differentially expressed miRNAs from each group were run for enrichment analysis using GO and KEGG enrichment, respectively, according to the correspondence between miRNA and its host gene. For more accurate results, the GOseq method was chosen to perform GO enrichment analysis. According to KEGG, pathway enrichment analysis was used to confirm the most crucial biochemical metabolic pathway and signal transduction pathway. Scores with *p* < 0.05 were considered noteworthy for enrichment analysis.

## 3. Results

### 3.1. Deep Sequencing of Sheep Pituitary Small RNA

**Figure 2:**
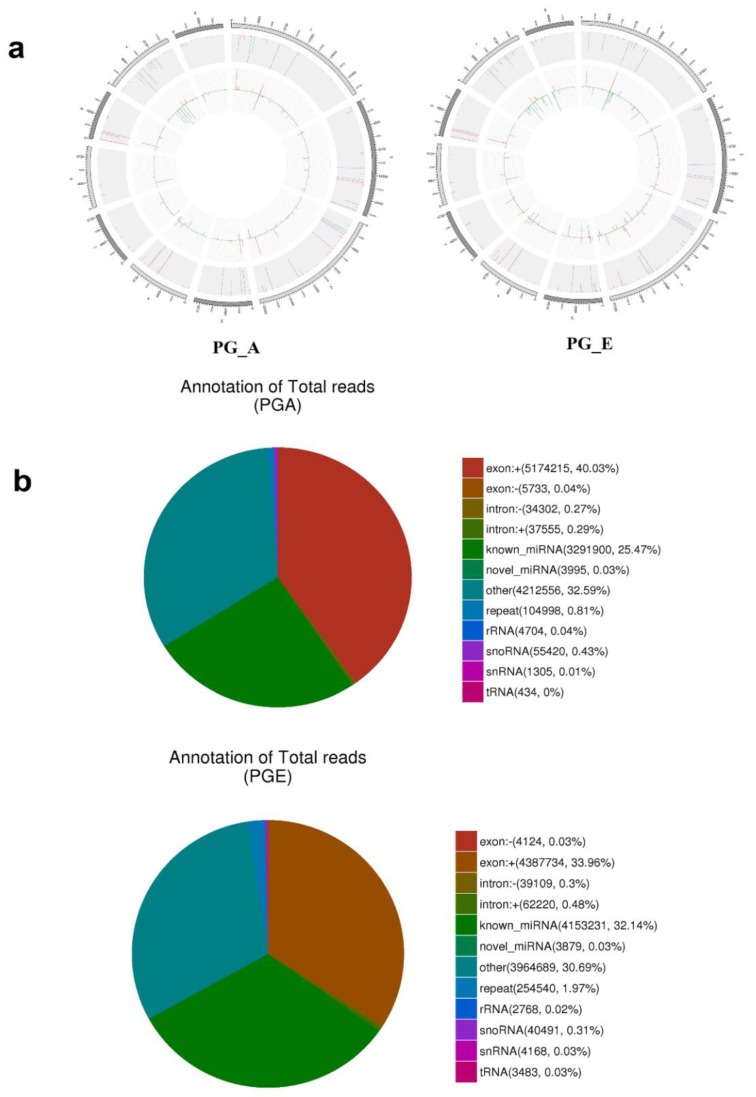
Distribution and annotation of miRNAs on chromosomes. (**a**) Distribution of miRNAs on chromosomes. Outermost circle is the chromosomes designated to display. Gray background region in the center is the distribution of 10,000 reads; red is mapped to the positive side chain; blue is mapped to the negative side chain; orange is positive chain distribution; and green is negative chain distribution. (**b**) Small RNA classification annotations.

To scientifically study the uniqueness and affluence of miRNAs, we scrutinized miRNA expression data from the pituitary gland in both estrus and anestrus states by Illumina HiSeq according to the flowchart (Figure 1a). Over 2 million raw readings have been found in the pituitary gland of sheep estrus and anestrus. The quality of the Illumina HiSeq data were reviewed and evaluated as it directly impacts subsequent bioinformatics analysis. We deleted the low quality readings and received 14,505,228 (PGA) and 14,256,069 (PGE) clean readings. Analysis of the sequence length of the sRNAs indicated that the majority of the length was 21 to 23 nucleotides (Figure 1b), similar to the common size of miRNAs. Bowtie software was used for the sequence alignment of sRNA reads to the reference sequence. Most of the sRNAs matched the reference sequence, accounting for 90.54% and 91.96% of the PGA and PGE library, respectively. A total of 192 mature miRNAs were mapped in miRBase. miRNAs expression in these two states of sheep pituitary is analogous (Pearson’s correlation coefficient PGE and PGA, r = 0.958) (Figure 1c). Study of these miRNA sources discovered that these miRNAs were dispersed on 26 autosomes and X chromosomes (Figure 2a). Moreover, we studied the efficiency of miRNAs obtained by high-throughput sequencing. Most of the sRNA sequences were clustered into miRNAs after alignment of the sRNAs with GenBank, the Rfam 11.0 database, small non-coding RNA, RNA repeat sequence, intron, and exon. In addition, we studied the alignment of these miRNAs. As a result, the ratios of exons, introns, intergenic regions, and rRNA constituting miRNAs in the PGA were 40.3%, 0.29%, 32.59%, and 0.04, respectively, while the ratios in PGE were 33.96%, 0.48%, 30.69%, and 0.02%, respectively (Figure 2b). However, this does not show a substantial difference in the arrangement of miRNAs between PGA and PGE but displays the least proportion of exons and intergenic regions in both conditions.

### 3.2. Analysis of Known and Novel miRNAs

**Figure 3:**
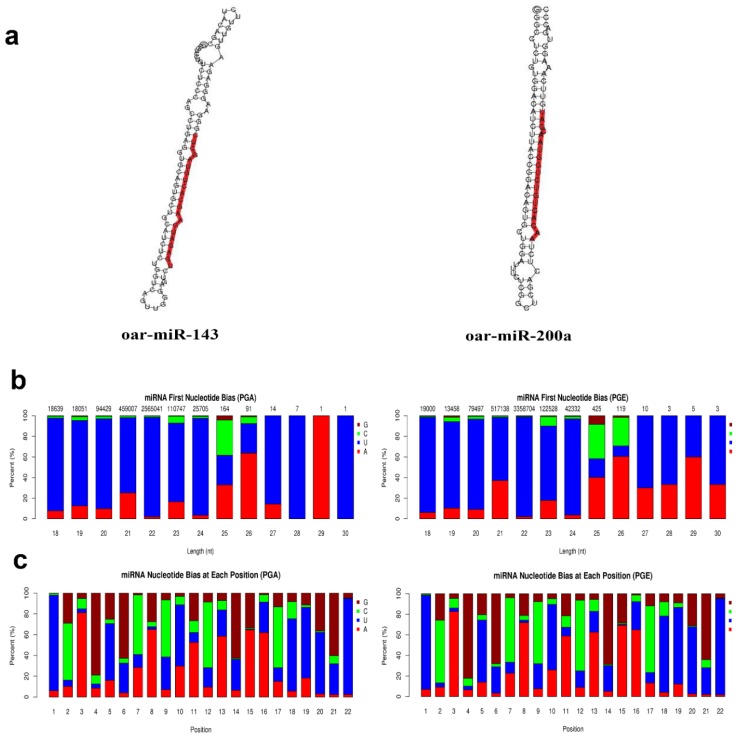
Analysis of known miRNAs. (**a**) Secondary structure of known miRNAs. The whole sequence is a precursor miRNA and the red highlight is mature sequence. (**b**) First base preference of known miRNAs of 18 to 30 nucleotides (nt) in length (the horizontal axis is the length of the miRNA and the vertical axis is the ratio of A/U/C/G in the first base of the miRNA in length). (**c**) Base distribution of known 22 nt miRNAs (horizontal axis is miRNA base position and the vertical axis is the ratio of bases A/U/C/G of the miRNA at this position).

**Figure 4:**
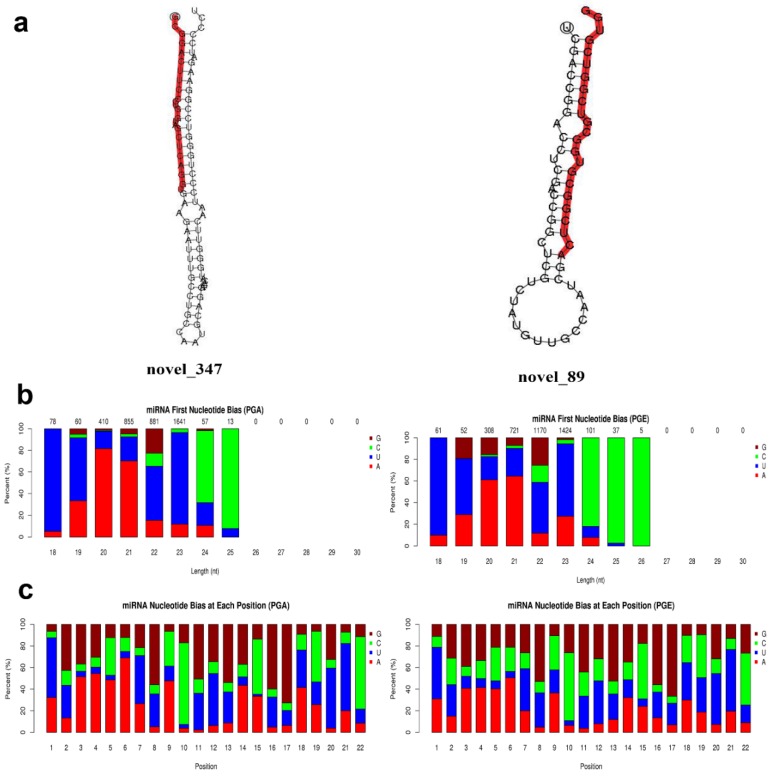
Novel miRNAs prediction and analysis. (**a**) Partially predicted novel miRNA secondary structure. The whole sequence is a precursor miRNA and red highlight is a mature sequence. (**b**) First base distribution of novel miRNAs 18~30 nt in length: the horizontal axis is the length of the miRNA, and the vertical axis is the ratio of A/U/C/G in the first base of the length sRNA (the value above the histogram is the length of the miRNA total number of items). (**c**) Base distribution of 22 nt novel miRNAs: the horizontal axis is the base position of the miRNA, and the vertical axis is the ratio of the bases A/U/C/G of the miRNA at this position.

Secondary structures of identified miRNAs were obtained by aligning the reference sequence with specific sequences in miRBase (Figure 3a). A total of 147 miRNAs were discovered, of which PGA comprises 145 miRNAs and PGE comprises 147 miRNAs. The progress of miRNA from the precursor molecule to the developed molecule is digested by Dicer. Due to restriction site specificity, the first base of a mature miRNA sequence is highly biased. Hence, we further analyzed the frequency distribution of the first base of miRNAs of different lengths and the frequency distribution of bases at various positions of the 22 nucleotides miRNAs (Figure 3b,c). These miRNAs were found to be concentrated in the sheep anestrus pituitary group (PGA) and the estrus pituitary group (PGE). The hairpin structure characteristic of miRNA precursors can be used to calculate novel miRNAs. We integrated miREvo and mirdeep2 software for analysis of new miRNAs (Figure 4a). A total of 52 novel miRNAs were found, of which PGA includes 47 novel miRNAs and PGE includes 47 novel miRNAs. Similarly, we analyzed the frequency distribution of the first base of novel miRNAs of different lengths and the frequency distribution of bases at various positions of the 22 nt novel mRNAs (Figure 4b,c).

### 3.3. Differential Analysis of Sheep Pituitary miRNAs 

**Figure 5:**
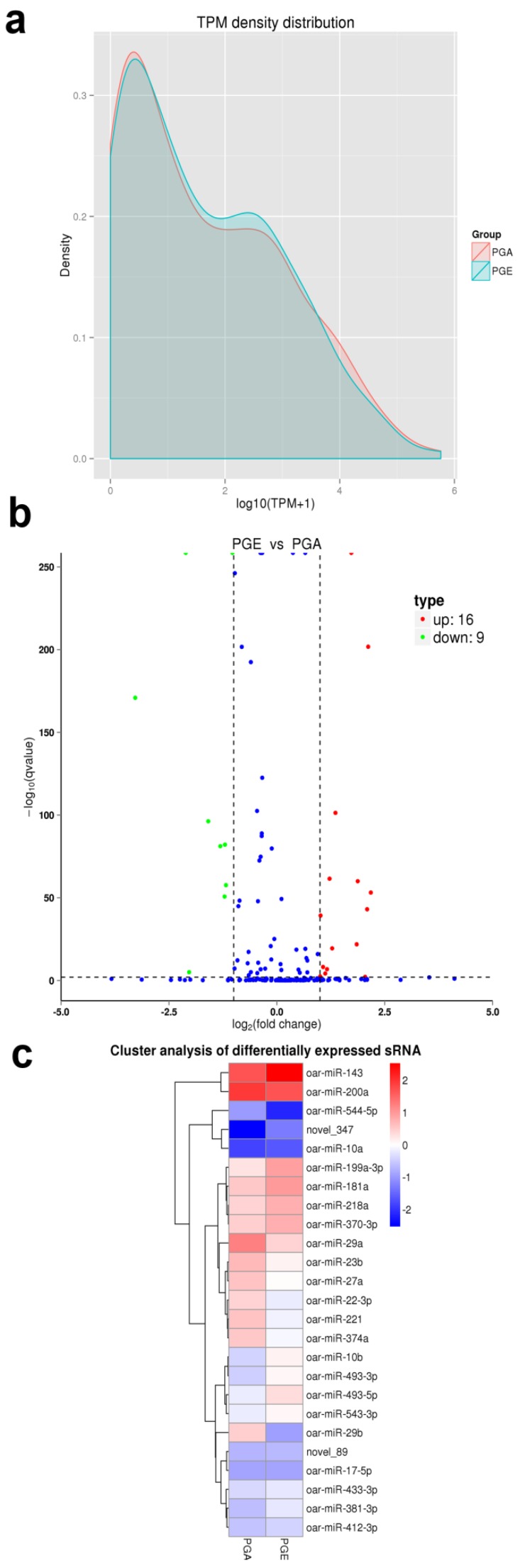
Analysis of differentially expressed miRNAs. (**a**) Density distribution transcripts per million (TPM) of miRNA expression levels. The horizontal axis is the log10 (TPM + 1) value of the miRNA, the vertical axis is the density corresponding to log10 (TPM+1), and different colors represent different groups. (**b**) Analysis of volcanic plot of miRNAs differentially expressed in sheep PGA and PGE. Scattered dots in the figure represent specific miRNAs, blue dots represent miRNAs with no major differences, red dots represent considerably upregulated miRNAs, and the green dots represent considerably downregulated miRNAs. (**c**) Cluster analysis of miRNAs differentially expressed in sheep PGE and PGA. Clustered with log10 (TPM + 1) values, red indicates high miRNA expression, and blue indicates low miRNA expression.

To understand the expression levels of both known and novel miRNAs in each sample, we used TPM to normalize expression levels and detect them by the density distribution of TPM (Figure 5a). A total of 25 differentially expressed miRNAs were found in PGA and PGE. This included 16 upregulated miRNAs and 9 downregulated miRNAs (Table 1, Figure 5b) (q-value < 0.01 and |log2 (fold change)| >1). Notably, it includes two novel miRNAs: novel_347 and novel_89. To further obtain the clustering model of two differentially expressed miRNAs, we accomplished a hierarchical clustering analysis using the TPM values of all the miRNAs combined (Figure 5c).

### 3.4. Confirmation of Differentially Expressed miRNAs

**Figure 6:**
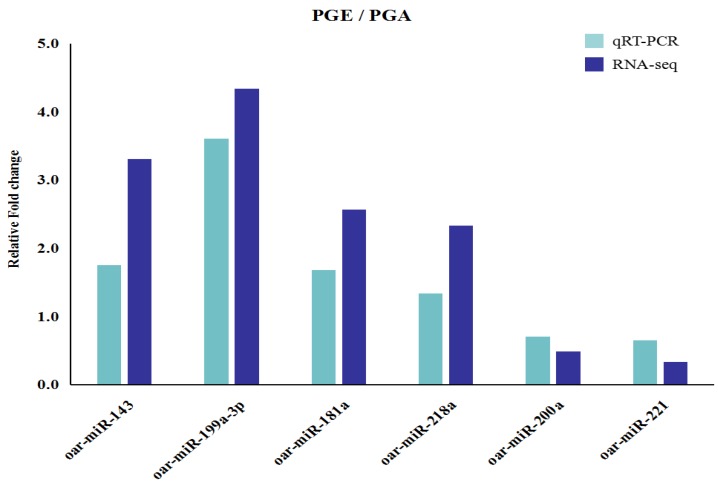
Validation of differentially expressed miRNAs by qRT-PCR. Six differentially expressed miRNAs expression levels as detected by qRT-PCR, and error bars indicate ± SD.

In order to verify the accuracy of the differentially expressed miRNA by RNA-seq data analysis, six differentially expressed miRNAs were randomly selected for RT-qPCR analysis and the amount of the selected miRNA was normalized to the amount of U6 snRNA. Analyzing the results, we found that the overall trend of this data was the same as our RNA-seq data and the results of the two methods were highly consistent. Compared to the PGA library, the results demonstrated that oar-miR-143, oar-miR-199a-3p, oar-miR-181a, and oar-miR-218a were upregulated, but oar-miR-200a and oar-miR-221 were downregulated (Figure 6). The data above indicate that the miRNAs we analyzed actually occurred in vivo, our RT-PCR data highly correlated with the sequencing data, and the high-throughput sequencing was reliable.

### 3.5. Target Prediction and Pathways Analysis

**Figure 7:**
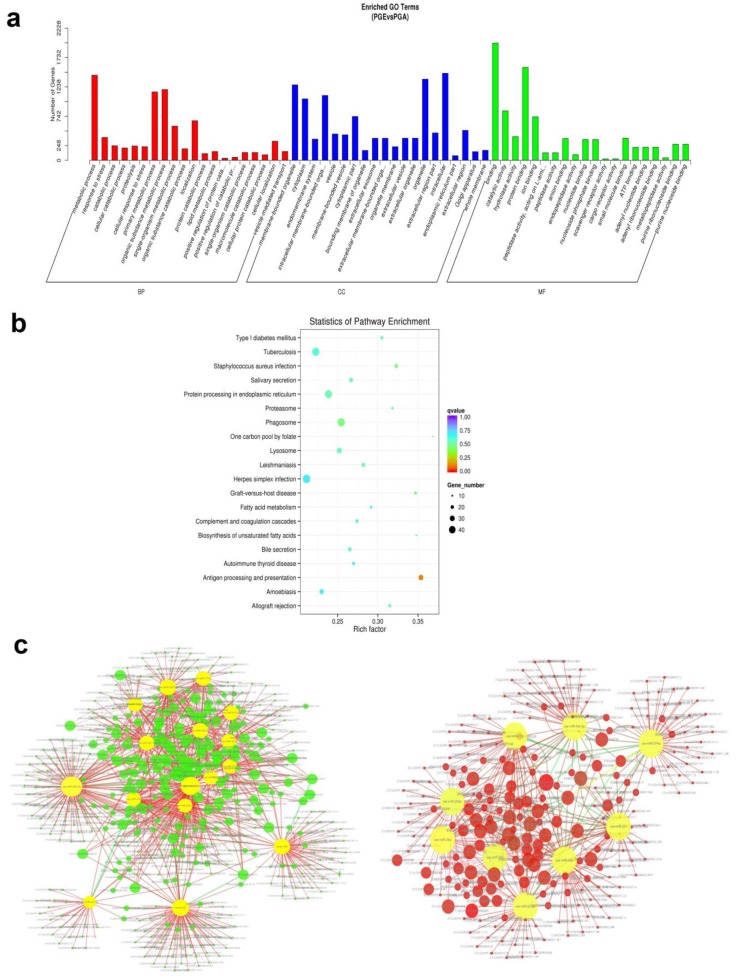
Enrichment analysis and annotation of miRNAs. (**a**) Gene ontology (GO) enrichment analysis of miRNAs differentially expressed in PGA and PGE (*p* < 0.05). Horizontal axis is the next level of GO polymer and GO terminology. Vertical axis is the ratio of genes annotated to the term. (**b**) miRNA differentially expressed host genes were subjected to Kyoto Encyclopedia of Genes and Genomes (KEGG) enrichment analysis (*p* < 0.05). The vertical axis signifies an access name and the horizontal axis signifies the access coefficient. Dots show the quantity of genes enriched during the visit. Colors are parallel to different q-values. (**c**) Regulatory networks of miRNA–mRNA. The left and right sides represent network regulation of up miRNA-down mRNA and down miRNA-up mRNA, respectively. Green and red circles represent target genes, and yellow circles represent upregulated miRNAs or downregulated miRNAs.

miRNA regulates biological processes by binding and interacting with targets. TargetScan and miRanda were used for the prediction of target genes for both known and novel miRNAs, and 3500 target genes were predicted (Table S2). To know the biological role of these differentially expressed miRNAs in a better way, GO analysis was used. We examined that a total of 153 GO terms (*p* < 0.05) were expressively enriched in three GO analysis categories (Table S3), comprising 28 molecular functions, 33 cellular components, and 92 biological processes. Interestingly, there is a very rich set of specific GO terms that are thoroughly related to expression and regulation of genes is greatly enriched, including those of the primary metabolism, the macromolecular metabolic process, the cell, the cellular part, the intracellular protein binding, and catalytic activity. Differentially expressed miRNA target genes are primarily associated with metabolic processes, and the cellular components are closely linked to cellular and organ functions (Figure 7a), suggesting that certain miRNAs may play a significant role in regulating pituitary gland functions. Next, the signaling pathways involved in these differentially expressed miRNAs were further analyzed using KEGG enrichment analysis. KoBas software showed that miRNAs that are differentially expressed are quite abundant in 272 signaling pathways (*p* < 0.05) (Table S4) and involved in metabolic regulation, disease, endocrine and cellular transmission, including amoebic disease and the lysosomal proteasome, which is linked to pituitary gland development and function (Figure 7b). Data above indicate miRNAs role in the pituitary gland. Furthermore, to carefully understand the role of miRNAs’ participation in sheep pituitary regulatory network, all the identified differentially expressed miRNAs in this report were projected for miRNA–mRNA binding by miRanda software. The general correlation between miRNA and mRNA with mapping of potential miRNA–mRNA regulatory networks was demonstrated by cytoscape 3.7.2 (Figure 7c).

## 4. Discussion

Although transcription studies have been performed on sheep genomes, to our knowledge this is the first report on miRNA expression profiles in sheep pituitary gland in the estrus and anestrus states, enriching information on the miRNA database in this animal species.

MiRNAs are a class of small non-coding RNAs. Different studies have revealed that miRNAs are closely linked to the growth and development of plants and animals. Carletti et al. found that miR-21 promotes the survival of the ovarian follicles during ovulation in mice [34]. In addition, let-7b in mice is involved in the normal development of corpus luteum [35]. Moreover, in the intestine, miR-143 plays a key role in colon cancer and post-cancer repair of intestinal epithelial injury; in particular, it may play an imperative role as a tumor suppressor and is probably contributed in metabolism. Previously, it was reported that miR-10a and miR-10b could remain associated with rapid remodeling of the duodenal mucosal epithelium in sheep. [18]. Likewise, miR-29a was reported as a major regulator of fibrotic cardiac and miR-21 was found to be increased in myocardial infarction. MiR-210-3p is closely linked to an inadequate oxygen supply at the tissue level. Remarkably, together miR-320a and miR-494 are associated with ischemia-induced cardiac apoptosis [19]. Numerous miRNAs have been submitted to miRBase for stress and digestion. In addition, 222 miRNAs have been described in the anterior pituitary of various pig species, and these miRNAs may adjust different growth circumstances in diverse pig species [36]. Nonetheless, recent studies have reported the identification of a lesser sum of new miRNAs in cattle (36 novel miRNAs), pigs (61 novel miRNAs), goats (35 novel miRNAs), and horses (329 novel miRNAs) [37]. Our study confirms the importance of miRNAs for animals. However, the role of miRNAs in sheep pituitary development is unclear. In this study, pituitary miRNAs involved in the regulation of sheep estrus were studied by Illumina HiSeq-technology. We detected a total of 29,116,686 raw reads by bioinformatics analysis. After removing low quality reads, we received clean reads: 14,256,079 in estrus and 14,505,228 in anestrus. Interestingly, we found that the majority of the length was 21–23 nt, which was found to be very similar to the size of typical miRNAs. Moreover, there were 199 differentially expressed miRNAs in PGA and PGE in this study, some of them were highly expressed in the reproductive stages of mammals, such as miR-26b and miR-200b, signifying that they may be extensively involved in sheep estrus and anestrus regulation. Some functions of these highly expressed miRNAs have been studied, for example miR-26b regulates the development of pituitary and growth hormone by targeting LEF-1 [12]. In mice, miR-200b promotes LH secretion by targeting transcriptional repressor ZEB1 (zinc finger E-box binding homeobox 1), which is associated with aggressive behaviour, metastasis, treatment resistance and poor prognosis in different tumour types, including breast, pancreatic and lung cancer [38,39]. These highly expressed miRNAs are thought to play key roles in the development of both pituitary and hormonal secretions and should be given more preference in the future. Furthermore, we identified 25 miRNAs that were differentially expressed in the PGA and PGE libraries. Compared to the PGA library, the PGE library had 16 upregulated miRNAs and 9 downregulated miRNAs. Next, several differentially expressed miRNAs were verified by RT-qPCR, and the results were constant with our sequencing results, indicating that these differentially expressed miRNAs may have significant roles in the pituitary gland. By predicting the targeted genes for these miRNAs, we found that certain targeted genes play an important role in both estrus and anestrus states. More specifically, we found a TRH gene linked to the development of the pituitary gland and to hormonal secretion in sheep estrus and anestrus by RNA-seq, which is targeted by miR-143, miR-29a, miR-29b, and miR-10a. A common target for miR-22-3p and novel_89 is the Kiss1 gene. Kiss1 and its GPR54 receptor execute a major role in the arousal and precocity of pituitary function [40,41,42]. Most miRNAs bind mRNA thereby regulating gene expression. In sum, our research suggests that these differentially expressed miRNAs are essential for pituitary functional development and animal reproductions. At the same time, novel_89 as a new differentially expressed miRNA is also considered to have important research value in the future. Recent studies have included several signaling pathways in the functional development of the pituitary gland [40,43]. However, little research has been done on miRNAs related to the functional development of sheep pituitary glands.

GO analysis specified that most of the differentially expressed miRNAs involved 28 molecular functions, 33 cellular components, and 92 biological processes. Simultaneously, analysis of the KEGG pathway reveals that miRNAs are involved in signaling pathways and in metabolic regulation, disease, endocrine, and cellular signaling that are directly linked to the pituitary function. Similarly, MAPK (mitogen-activated protein kinase) signaling pathway involves a variety of signaling cascades, of which Ras-Raf-Mek-ERK1/2 (MAPK / ERK) is one of the most dysregulated in human cancers. It has an important regulatory role in keeping the balance between Sox2 + pituitary cell proliferation and differentiation [44]. In addition, miRNA–mRNA pairs have been speculated and a complex network of interactions may be formed, but their mechanism of action remains unclear.

## 5. Conclusions

In conclusion, this study provides valuable information about miRNAs biology and aids us to comprehend miRNAs role in regulating basic biological developments like pituitary development. However, further experimentation is required to carefully evaluate the purpose of miRNAs and their predictive target analysis.

## Figures and Tables

**Table 1:** Differentially regulated miRNAs identified between PGE and PGA.

Gene_ID	PGE	PGA	log2.Fold_change.	q-Value	Corrected *p*-Value	UP/DOWN Regulate
oar-miR-143	32670.824	9873.594248	1.7264	0	0	UP
oar-miR-200a	8144.9197	16627.14523	−1.0296	0	0	DOWN
oar-miR-29a	923.39904	3991.937194	−2.1121	0	0	DOWN
oar-miR-199a-3p	2190.8655	504.9304155	2.1173	1.02 × 10^−203^	1.69× 10^−202^	UP
oar-miR-29b	85.951935	836.0509809	−3.282	9.42 × 10^−173^	1.21× 10^−171^	DOWN
oar-miR-181a	2319.1442	904.6077302	1.3582	4.40 × 10^−103^	4.58× 10^−102^	UP
oar-miR-221	327.96766	986.8189359	−1.5892	5.91 × 10^−98^	5.79× 10^−97^	DOWN
oar-miR-23b	520.64541	1197.040669	−1.2011	8.90 × 10^−84^	7.41× 10^−83^	DOWN
oar-miR-27a	426.90327	1058.504832	−1.31	7.82 × 10^−83^	6.20× 10^−82^	DOWN
oar-miR-218a	1721.6354	738.194044	1.2217	4.35 × 10^−63^	2.89× 10^−62^	UP
oar-miR-493-5p	780.5786	212.7819441	1.8752	1.55 × 10^−61^	9.90× 10^−61^	UP
oar-miR-374a	379.12334	858.2394724	−1.1787	3.64 × 10^−59^	2.24× 10^−58^	DOWN
oar-miR-10b	549.72884	121.4677676	2.1781	1.17 × 10^−54^	6.98× 10^−54^	UP
oar-miR-22-3p	314.72431	728.5221375	−1.2109	3.23 × 10^−52^	1.85× 10^−51^	DOWN
oar-miR-493-3p	470.78809	110.3735218	2.0927	1.80 × 10^−44^	8.81× 10^−44^	UP
oar-miR-370-3p	1608.4178	797.0788868	1.0128	1.16 × 10^−40^	5.52× 10^−40^	UP
oar-miR-381-3p	287.19891	79.65099512	1.8503	3.02 × 10^−23^	1.36× 10^−22^	UP
oar-miR-543-3p	487.66687	200.549827	1.2819	8.80 × 10^−21^	3.76× 10^−20^	UP
novel_347	42.846131	0	6.2348	4.85× 10^−11^	1.58× 10^−10^	UP
oar-miR-433-3p	287.45859	136.8290309	1.071	2.14× 10^−9^	6.84× 10^−9^	UP
oar-miR-412-3p	201.24698	89.89183734	1.1627	4.97× 10^−8^	1.50× 10^−7^	UP
oar-miR-544-5p	8.8288997	36.12741564	−2.0328	3.47× 10^−6^	9.64× 10^−6^	DOWN
novel_89	130.61578	60.02271418	1.1217	2.29× 10^−5^	5.96× 10^−5^	UP
oar-miR-17-5p	92.443773	46.08379003	1.0043	0.001524	0.0038437	UP
oar-miR-10a	22.331923	5.404888954	2.0468	0.0027333	0.0067909	UP

## Supplementary Materials

The following are available online at https://www.mdpi.com/2076-2615/10/3/402/s1. Table S1: Primers in this study for Quantitative real-time PCR, Table S2: information of known and novel miRNAs, Table S3: GO analysis of differentially expressed miRNAs, Table S4: KEGG pathway analysis enriched in 272 terms.
